# Antitumor effects of different administration sequences of cisplatin and Endostar on Lewis lung carcinoma

**DOI:** 10.3892/ol.2014.2783

**Published:** 2014-12-09

**Authors:** JUAN FAN, JIANGRONG DU, JINGBO WU, SHAOZHI FU, DEFENG HU, QIANG WAN

**Affiliations:** 1 Department of Oncology, The Affiliated Hospital of Luzhou Medical College, Luzhou, Sichuan 646000, P.R. China; 2 Department of Nuclear Medicine, Luzhou Medical College, Luzhou, Sichuan 646000, P.R. China

**Keywords:** Endostar, cisplatin, anti-angiogenesis, drug concentration, combined therapy

## Abstract

Angiogenesis plays an essential role in the growth and metastasis of a number of tumors. Anti-angiogenic drugs are able to normalize tumor vasculature and inhibit tumor growth. Therefore, it has been hypothesized that the combination of cytotoxic chemotherapy drugs and angiogenesis inhibitors may exert complementary therapeutic benefits in the treatment of cancer. In the present study, the effect of the angiogenesis inhibitor, recombinant human endostatin (Endostar), in combination with cisplatin, was evaluated in C57/BL/6 mouse xenografts under different administration sequences. The drug combinations and sequences of administration were analyzed within the cancer xenografts for any inhibitory effects. Changes in the cell cycle distribution of the cells were monitored using flow cytometry. The effects of Endostar, particularly a reduction in the density of microvessels, were assessed using a method that employed anti-cluster of differentiation 31 antibodies. The concentration of cisplatin in the blood and tumor tissue at various time-points following administration was detected by high-performance liquid chromatography. The tumor tissues that received simultaneous Endostar and cisplatin exhibited increased inhibition of tumor growth and improved cell cycle distribution compared with those that received cisplatin alone, or those in which Endostar was administered prior to cisplatin. The simultaneous administration of the drugs resulted in the lowest microvessel density in the xenografts. Under these conditions, the concentration of cisplatin was revealed to be the highest in the grafted tumor tissue. The results of the present study suggest that the co-administration of Endostar and cisplatin may aid in the optimization of the antitumor activity of cisplatin.

## Introduction

Lung cancer is one of the most commonly diagnosed malignant tumors in China. Non-small cell lung cancer (NSCLC) demonstrates the highest incidence and accounts for 80% of all lung cancer cases. Lung cancer exhibits a high rate of mortality and is often not diagnosed at an early stage. Therefore, 75% of NSCLS patients are ineligible for surgical resection at the time of diagnosis (1). The principal therapeutic modality for the treatment of advanced-stage lung cancer is chemotherapy, which usually consists of a combination of third-generation cytotoxic drugs and platinum (2). However, a limitation of this therapy is a reduction in the quality of life of the patients due to the side-effects of the drugs. In addition, drug resistance is common in cases of NSCLC, and therefore relapse rates are extremely high.

The growth and metastasis of a tumor depends upon the steady supply of nutrients and oxygen, which are delivered via the vascular system. All solid malignant tumors develop a network of blood vessels. For this reason, anti-angiogenic ‘hunger therapies’ are used to limit the development of the blood vessels (3). Anti-angiogenic therapies are able to work synergistically with conventional chemotherapy treatments (4–6). However, certain studies have reported negative results with the combined use of anti-angiogenic and chemotherapy drugs (7, 8). Hypotheses, such as the ‘time window’ (9) and ‘vascular normalization’ (10), have been proposed in order to explain these negative findings. A study by Weichselbaum (11) revealed that γ-ray treatment of U87-MG xenografts was more effective in decreasing tumor size when the tumors were treated with the vascular endothelial growth factor inhibitor, DC101, for 4–6 days prior to radiation treatment (11). Other studies suggested that anti-angiogenic drugs combined with chemotherapy may exhibit optimal efficacy when administered successively, and that a short ‘time window’ for optimal results may exist (12–14). However, certain evidence exists that contradicts this hypothesis (15–17). Therefore, further research to address which optimal combination and administration regime of anti-angiogenic and antitumor drugs, whether it may be simultaneous or sequential, is required.

Cisplatin, a non-specific cell cycle-dependent agent, is the primary chemotherapeutic drug used to treat cases of NSCLC. The recombinant human endostatin, Endostar, is the first anti-angiogenic drug to be developed in China, and has been reported to be more efficient in blocking angiogenesis and suppressing the growth of primary tumors and metastases compared with other endostatin preparations (18). Endostar, in combination with vinorelbine and cisplatin, has been approved for use in the treatment of advanced NSCLC (19). The present study aimed to identify the optimal treatment regimen for the combination of Endostar and cisplatin in a murine tumor model, and to define a treatment schedule in order to guide future clinical therapies more efficiently than existing protocols.

## Materials and methods

### Experimental animal and tumor models

C57/BL/6 mice, 6–8 weeks old, were purchased from Tengxin Biotechnology Co., Ltd., (Chongqing, China) and housed in the animal research facility at The Affiliated Hospital of Luzhou Medical College (Luzhou, China). The mice were kept in groups of between three and five animals per cage, and fed with clean food and water. The animals were acclimatized to laboratory procedures for at least a week under the standard conditions of 24±2°C and 50±10% relative humidity. The murine Lewis lung carcinoma (LLC) cell line was obtained from The Experimental Center of Sichuan University (Chengdu, China) and maintained in RPMI-1640. In total, ~1×10^6^ LLC cells were suspended in 0.1 ml phosphate buffered saline (PBS; pH, 7.0) and then injected subcutaneously into the right lumbar region of each mouse. Following the development of tumors, the tumor tissue blocks were resected and then implanted into the right lumbar region of another mouse. The tumor cells were passaged in this way for three generations in order to adapt them to the *in vivo* environment. The tumor growth was evaluated every other day by the measurement of the tumor diameter. The volume of each tumor was determined using the following formula: Tumor volume (cm^3^) = length × width^2^ × 0.5. All animal studies were approved by the Institutional Animal Care and Treatment Committee of Luzhou Medical College.

### Chemotherapy

Cisplatin was purchased from Gejiu Bio-Medicine Industry Ltd. (Yunnan, China). According to the dose conversion table for animal and human body weights, which uses the Du Bois formula to calculate the body surface area (BSA) of the patient (m^2^): 0.007184 × (patient height in cm)^0.725^ × (patient weight in kg)^0.425^, the maximum daily dose of cisplatin for mice is 6.15 mg/kg. Therefore, doses of 6, 5, 4 and 3 mg/kg/day were selected for the preliminary experiments. Cisplatin was administered intraperitoneally (i.p.). At 4 mg/kg/day, cisplatin exerted the maximum antitumor effect, therefore, this dose was selected for use in the combination study with Endostar, which was diluted in 0.2 ml sterile 0.9% normal saline (NS).

### Design and grouping of experiments

**Figure 1:**
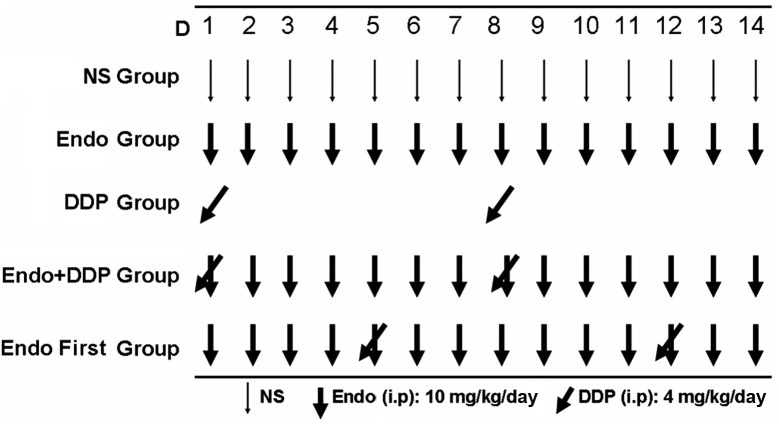
Treatment schedule. One week after inoculation, the mice were randomized to five different treatment groups and treated with either normal saline (NS), Endostar, cisplatin, Endostar + cisplatin or Endostar followed by cisplatin (Endostar first), as described in the Materials and methods section. I.p, intraperitoneally; DDP, cisplatin.

The recombinant human endostatin, Endostar, was provided by Shandong Simcere Medgenn Biopharmaceutical Co., Ltd (Yantai, Shandong, China) and stored at 4°C until required. According to the protocols adopted in the preliminary experiments, Endostar was dissolved in 0.2 ml 0.9% NS and administered to each animal at a dose of 10 mg/kg/day. All drugs were administrated via i.p. injection according to the regimen shown in Fig. 1. When the diameter of the tumors had reached between 8 and 10 mm, the animals were randomized into five groups (n=7 in each group) and treated for 14 consecutive days as follows: i) NS group (negative control); ii) Endostar group (10 mg/kg/day); iii) cisplatin group (4 mg/kg/day administered on days 1 and 8); iv) Endostar + cisplatin group (drugs administered simultaneously according to the same dose and regimen as the Endostar and cisplatin groups; and v) Endostar first group (Endostar administered on days 1 to 14 and cisplatin administered on days 5 and 12). In order to assess the tumor growth inhibition rate and analyze any histological changes, the animals were sacrificed on day 15 by cervical dislocation. The tumors were then excised and weighed. A section of each tumor was fixed in 10% neutral formaldehyde solution in preparation for the histological analysis, and another section was fixed with 70% ethanol for the flow cytometry analysis. The tumor growth inhibition rate was determined using the following formula: Inhibition rate (%) = (1 − Wep/WNS) × 100, where Wep is the average tumor weight of the experimental group, and WNS is the mean tumor weight of the control group. The co-operation index (q) was calculated as follows: q = Ea + b/Ea + Eb − Ea × Eb, where Ea + b represents the inhibition rate of the drugs combined, and Ea and Eb represent the inhibition rate of the drugs used alone, respectively. In order to measure the cisplatin serum and tissue concentrations, the animals were randomized into three groups (n=20 in each group) as follows: i) The cisplatin group; ii) the Endostar + cisplatin group; and iii) the Endostar first group. Each group was treated using the same doses and regimens as used in the aforementioned experiment. Blood samples were collected by retro-orbital bleeding on days 1 (20 min after the first injection of cisplatin), 3, 5 and 8 (20 min after the second injection of cisplatin). The mice were then sacrificed and the collected blood samples and excised tumors were stored in preparation for the high-performance liquid chromatography (HPLC) analysis. A section of tumor tissue was also fixed using 10% neutral formaldehyde for use in the immunohistochemical analysis.

### Flow cytometry analysis

The tumor tissues were fixed with 70% cold ethanol and prepared into a single cell suspension. Next, the suspension was precipitated following centrifugation at 95 × g for 10 min. The cells were then rinsed twice in PBS (pH 7.4) for 10 min each. Next, the samples were stained with propidium iodide (Beckman Coulter Inc., Brea, CA, USA) for the cell cycle analysis. The proliferation index (PI) was calculated using the following equation: PI = 100 × (S + G_2_M)/(G_0_/G_1_ + S + G_2_M).

### Immunohistochemistry

The tumors were fixed in 10% neutral formaldehyde, embedded in paraffin and then cut into 3-μm sections. Next, the sections were stained with hematoxylin and eosin. The sections were then labeled with a rabbit anti-mouse anti-cluster of differentiation 31 (CD31) antibody (1:10 dilution; Bio-World, Dublin, OH, USA) and visualized using a biotinylated goat anti-rabbit anti-immunoglobulin G (IgG; 1:10 dilution) and streptavidin peroxidase (SP)-conjugated antibody (1:10 dilution; Bio-Rad Laboratories, Inc., Hercules, CA, USA) to assess the proliferation of microvessels. The sections were deparaffinized in xylene and then rehydrated in a graded alcohol series as follows: Xylene for 10 min (x2), 95% ethanol for 5 min (x2) and 100% ethanol for 5 min (x2). Following high pressure saturated steaming (126°C, 1.6 bar, 23 psi), antigen retrieval was performed by autoclaving the tumor sections in retrieval buffer (EDTA buffer, pH 6.0) in the saturated steam for 10 min. The endogenous peroxidase activity was blocked with 3% hydrogen peroxide in methanol for 10 min at room temperature. Next, the sections were rinsed twice with PBS and then incubated with the rabbit anti-mouse anti-CD31 (1:10 dilution) antibody for 1 h at room temperature, followed by incubation with the biotinylated goat anti-rabbit anti-IgG antibody [diluted 1:2,000 in BLOTTO (Thermo Fisher Scientific, Waltham, MA, USA]) at room temperature for 1 h and staining with 3,3′-diaminobenzidine for brown color development. Quantification of the microvessel density (MVD) was performed according to the method used by Weidner *et al* (20). First, the sections were screened at a low magnification (×100) in order to identify the region of the tumor that exhibited the densest vascularization, designated as a ‘hot spot’. Within the hot spot, stained microvessels were counted in a single high-power field (×400). The MVD was expressed as the number of microvessels counted per field. Any CD31-stained endothelial cells, or endothelial cell clusters that were clearly separated from adjacent microvessels, tumor cells or connective tissue were considered to be a single microvessel.

### HPLC

In order to measure the cisplatin concentration within the tumors, the tumor tissue was homogenized and centrifuged at 16,060 × g for 10 min. The homogenate was then collected, and 500 μl methanol was added to the cell suspension. In order to measure the plasma concentration of cisplatin, 500 μl methanol was added to the blood plasma to precipitate the proteins. Next, the suspension was centrifuged at 16,060 × g for 10 min, and 50 μl 5% DDTC was added. The mixtures were then incubated at 37°C for 30 min. The protein precipitate was extracted using 500 μl chloroform, vortexed for 2 min and then centrifuged at 16,060 × g for 15 min. The chloroform phase was transferred to another vial and evaporated under dry air. The precipitate was then re-dissolved in 100 μl chloroform and vortexed for a further 30 sec. In total, 15 μl precipitate was injected into the HPLC device. The HPLC device (Ultimate 3000; Dionex, Sunnyville, CA, USA) was equipped with an Ultimate 3000 array detector, which uses UV light at 254 nm at room temperature and a narrow-bore column (Hypersil C18 column; 250×4.6 mm; 5-μm particle size). The mobile phase used methanol/water (75/25, v/v) at a flow rate of 0.1 ml/min. A standard curve was used for the quantification of cisplatin in the blood and tumor tissue.

### Statistical analysis

All data are expressed as the mean ± standard deviation. Comparisons between multiple groups were performed using a one-way analysis of variance, followed by Dunnet’s test. The statistical analyses of the results were performed using SPSS software version 19.0 (SPSS, Inc., Chicago, IL, USA). P<0.05 was considered to indicate a statistically significant difference.

## Results

### Effects of cisplatin and Endostar on LLC tumors

**Figure 2:**
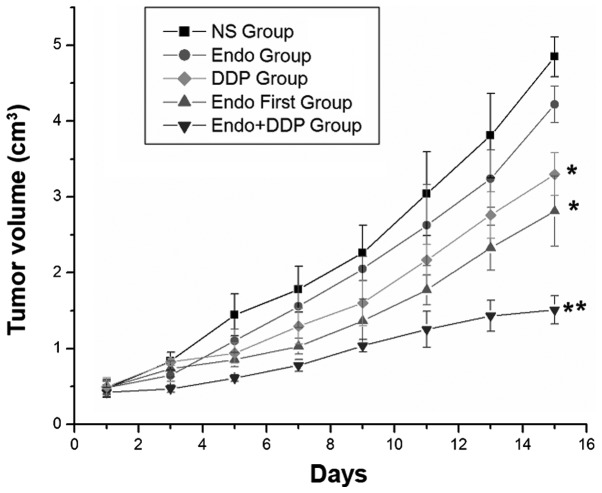
Graph revealing the mean tumor volumes on alternate days for each treatment group. ^*^P<0.05 vs. the normal saline (NS)-treated group; ^**^P<0.05 vs. the Endostar first-treated group. DDP, cisplatin.

**Figure 3:**
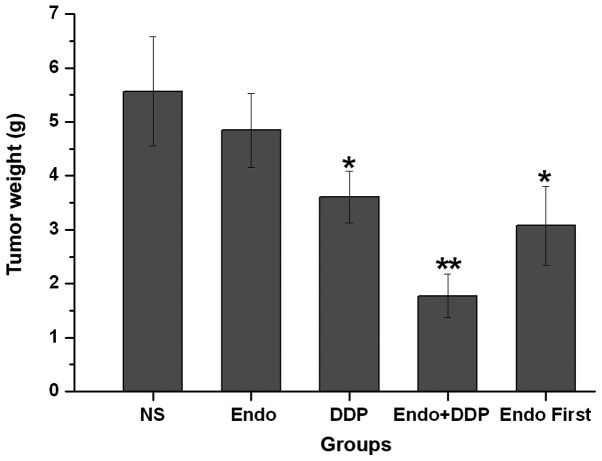
Mean tumor weights of the mice from each group following different treatment schedules. ^*^P<0.05 vs. the normal saline (NS)-treated mice; ^**^P<0.05 vs. the Endostar first-treated group. DDP, cisplatin.

In order to determine the optimal treatment regimen, the present study investigated the effect of the combination of Endostar and cisplatin on the growth of LLC tumors in C57/BL/6 mice. Treatment with cisplatin demonstrated significant inhibition of tumor growth compared with NS or Endostar alone. Compared with all other groups, the cisplatin + Endostar group demonstrated the most significant inhibition of tumor growth, as determined by tumor volume and weight on day 14 (P<0.001). This comparison included the Endostar first group, which also received a cisplatin and Endostar combination treatment, but one in which the Endostar treatment was started prior to the cisplatin treatment (Figs. 2 and 3). The tumor growth rate was significantly lower in the cisplatin, cisplatin + Endostar and Endostar first groups compared with the control group. The q index of the cisplatin + Endostar group was 1.564, which indicated a synergetic effect when the drugs were administered simultaneously. However, the q value of the Endostar first group was 1.095, which suggested that the effects of the drugs were additive when administered successively.

### Effects of cisplatin and Endostar on the distribution patterns of the cell cycle

**Figure 4:**
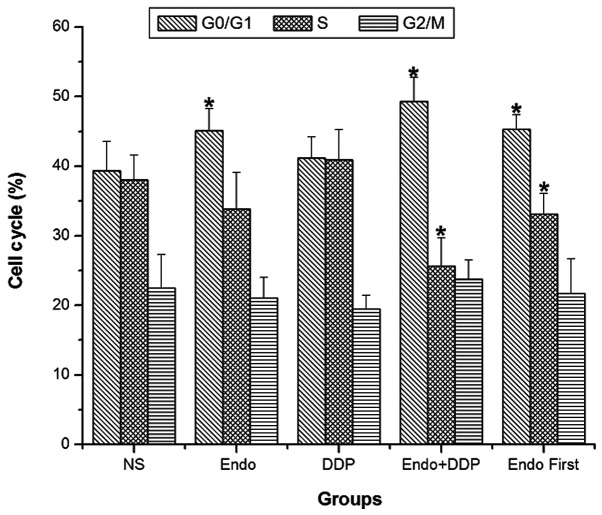
Mean cell cycle distribution following different treatment schedules for 14 days. ^*^P<0.05 vs. the normal saline (NS)-treated mice. DDP, cisplatin.

After 14 days of treatment, cell cycle analysis of the tumor cells was performed using flow cytometry. The PIs were calculated for each treatment group as described in the Materials and methods section. The PI values of the NS, Endostar, cisplatin, Endostar + cisplatin and Endostar first groups were 60.514±4.245, 55.600±4.494, 60.371±5.033, 49.386±2.149 and 54.386±2.812, respectively. The proportion of tumor cells in the G_0_/G_1_ phase was significantly higher in the Endostar, cisplatin + Endostar and Endostar first groups compared with the control group. These groups also demonstrated a decreased PI compared with the control group. The difference in the proliferation index was most significant when the two drugs were administered simultaneously (P<0.05). The PI of the cisplatin + Endostar group was significantly lower compared with that of the Endostar first group (P<0.05) (Fig. 4).

### Inhibition of tumor-induced angiogenesis

**Figure 5:**
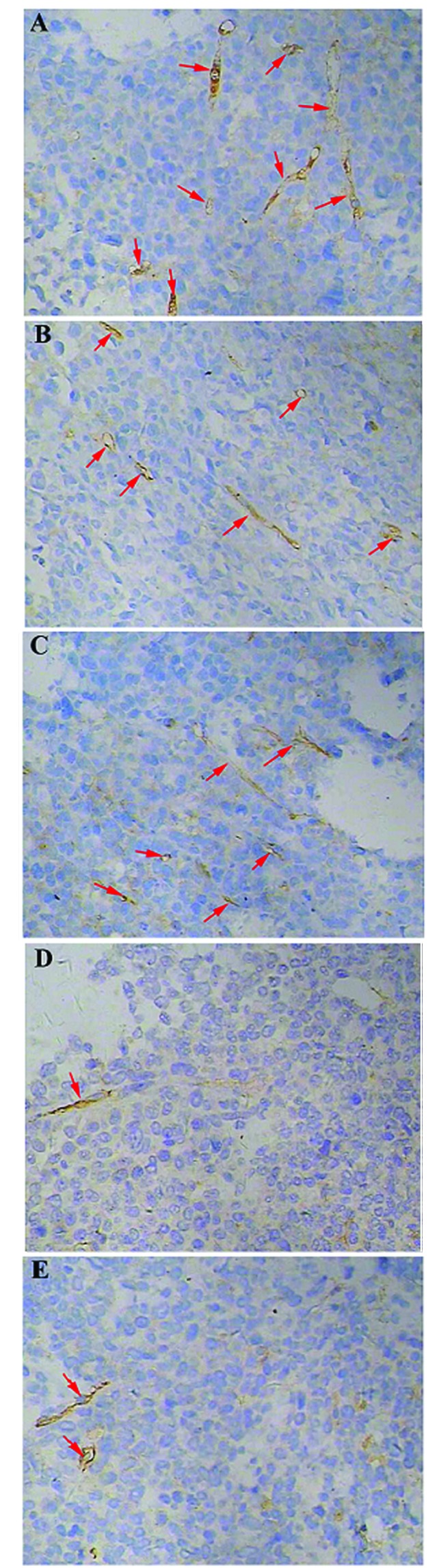
Immunohistochemical analysis revealing new vascular endothelial cells (magnification, ×400). Cluster of differentiation 31-stained endothelial cells and endothelial cell clusters were brown and clearly separated from adjacent microvessels, tumor cells or connective tissue elements. A) NS group, B) Endostar group, C) cisplatin group, D) Endostar + cisplatin group and E) Endostar first group. Red arrows indicate the formation of new blood vessels.

**Figure 6:**
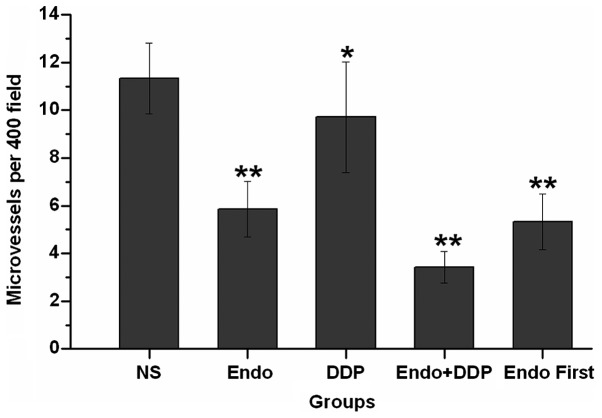
Quantification of mean microvessel densities of the tumor tissues following different treatment schedules for 14 days. ^*^P<0.05 vs. the normal saline (NS)-treated mice; ^**^P<0.01 vs. the DDP groups. DDP, cisplatin; Endo, Endostar.

Angiogenesis within the tumor tissues was estimated by the measurement of the MVD, which was performed by counting the number of microvessels in a given area. CD31 was used as a marker of microvessels within the tumor tissues. The tumor microvessel count in the control group was higher than that in all other groups. The difference in the MVD was particularly notable when comparing the tumors from animals simultaneously treated with cisplatin and Endostar with those in the control group (P<0.05). Furthermore, the microvessel densities in the animals from the simultaneously treated group were lower than those observed in the Endostar first group (P=0.21) (Figs. 5 and 6).

### Tumor tissue and blood concentrations of cisplatin

The cisplatin concentrations in the blood and tumor tissue were measured by HPLC at various time-points. The HPLC analysis revealed a cisplatin peak in the blood at 10 min, which was distinct from an endogenous impurity peak. The cisplatin concentration in the blood was higher than that in the tumor tissue in all groups (P<0.05). Overall, no statistically significant differences were identified between the cisplatin concentrations of the three groups on the first day. On day 3, the group that received the two drugs simultaneously exhibited higher cisplatin concentrations in the tumor tissue than in the blood (P<0.05). This was not observed in the other drug-treated groups. Similar results were observed on day 5 (P<0.05). On day 8, the cisplatin concentrations in the blood and tumor tissues increased significantly in all groups when compared with the earlier time-points. There were no statistically significant differences identified in the blood concentrations between the groups (P>0.05). As on days 3 and 5, the concentration of cisplatin in the tumor tissues from the mice simultaneously treated with cisplatin and Endostar was higher than that in the blood. The cisplatin concentrations were also higher in the blood than in the tumor tissues in the Endostar first and cisplatin alone groups (P>0.05). The cisplatin concentrations in the blood and tumor tissue in the Endostar first and cisplatin groups were not statistically different (Table I). The MVD in the tumor tissues from the three drug-treated groups demonstrated an association with the concentration of cisplatin. Overall, there were no statistical differences identified in the MVD between the three groups on the first and third days of treatment. However, the MVD of the tumor tissue from the simultaneously-treated group was lower than that of the cisplatin-only group on day 5 (P>0.05). On day 8, the MVDs of the combination drug groups were lower compared with the cisplatin-only group (P>0.01) (Table II).

## Discussion

The growth of primary tumors and metastases depends upon a network of blood vessels that receive nutrients and oxygen. Preclinical and clinical studies have revealed that anti-angiogenic agents are able to normalize the tumor vasculature (21–23). The identification of the optimal ‘time window’ of treatment is a key factor to ensure the normalization of blood vessels. An excessive or prolonged treatment period with anti-angiogenic drugs can damage and degrade normal blood vessels. Weichselbaum (11) estimated the duration of this optimal time window of treatment to be between four and six days. A study by Peng *et al* investigated whether Endostar could create a ‘vascular normalization window’ to alleviate hypoxia and enhance the inhibitory effects of radiation therapy in human nasopharyngeal carcinoma xenograft models (24). The results of the study revealed that when Endostar was administered for three or five days, it alleviated hypoxia and significantly sensitized the tumor tissue to radiation. These results suggested that the optimal time window for Endostar treatment combined with radiation was between three and five days, which was consistent with the results described by Weichselbaum (11) Another study reported an optimal treatment time of between four and six days for Endostar combined with chemotherapy (25). Therefore, in the present study the mice were first treated with Endostar for four days prior to initiating cisplatin treatment in the group that received Endostar and cisplatin in succession. However, the successive treatment of cisplatin and Endostar was not as effective as the simultaneously administered combination. Although the sequential administration of cisplatin and Endostar exhibited increased tumor inhibition compared with the tumors treated with Endostar or cisplatin alone, the sequential treatment was less effective than the simultaneous treatment and demonstrated only additive effects of the drugs.

The results of the present study suggest that the combination of cytotoxic chemotherapy agents and angiogenesis inhibitors may produce a synergistic therapeutic effect in the treatment of cancer. When combined with chemotherapy, anti-angiogenic therapy could result in the normalization of the vascular structure and in the inhibition of tumor growth. The hydrostatic pressure of the tumor tissue could potentially be lowered by the anti-angiogenic agents, which would allow the chemotherapy drugs to easily enter the core of the tumor tissue. Another proposed mechanism is that a reduction in the extent of angiogenesis in solid tumors creates a hypoxic environment, which causes the tumor cells to become more sensitive to the chemotherapy treatment (26). In the present study, the concentration of cisplatin in the blood and tumor tissues was measured by HPLC, and the MVD was analyzed using immunohistochemistry. The MVD is the most common indicator used to measure the degree of angiogenesis, and is correlated with the metastatic activity of tumors (27). The results of the present study revealed that the combination of Endostar and cisplatin resulted in a higher cisplatin concentration in the tumor tissue. The synergistic administration of Endostar and cisplatin also resulted in a reduced MVD. Cytotoxic drugs have been demonstrated to effectively inhibit the formation of blood vessels over a large dose range (28). The MVD of the cisplatin-treated mice was significantly lower than that of the NS-treated mice (P=0.048) in the present study. In theory, if angiogenesis inhibitors are active along with chemotherapy drugs, the two can produce a synergistic anti-angiogenic effect. However, the curative effect observed in the Endostar first group was less than that in the Endostar + cisplatin group. Therefore, it is likely that treatment with Endostar resulted in partial normalization of the tumor vasculature in the first four days of treatment, and that the cisplatin administered thereafter may have initiated degeneration of the vascular network due to its own anti-angiogenic effects.

In the clinical setting, cell cycle analyses are used to guide the treatment of malignant tumors. Studies have revealed that Endostar acts at a slower rate on tumor tissues than cytotoxic drugs, and is predominantly effective in the G_0_/G_1_ phase of the cell cycle where it induces cellular apoptosis (29,30). The rate of apoptosis is usually increased when Endostar is administered in combination with chemotherapy drugs (16,31). As a non-specific cytotoxic drug, cisplatin has exhibited a minimal effect upon the proportion of cells in a specific phase of the cell cycle. However, when combined with Endostar in the present study, cisplatin synergistically altered the cell cycle distribution of the tumor cells. This drug combination increased the number of cells undergoing G_0_/G_1_ cell cycle arrest, decreased the number of cells undergoing G_2_/S arrest and lowered the PI. When the two drugs were used simultaneously, the synergistic effect was more notable than when the drugs were administered sequentially.

The present study did not determine an optimal time window of treatment for the co-administration of cisplatin with Endostar. Instead, the results demonstrated that cisplatin and Endostar may exhibit synergistic effects, which prevent the proliferation of solid tumors by reducing the density of microvessels and allowing greater penetration of cisplatin into the tumor tissue. This may be due to the fact that traditional cytotoxic drugs exhibit an anti-angiogenic effect at low doses. Following treatment with Endostar for four days, a region of the tumor vascular became normalized. The subsequent addition of cisplatin may have lead to excessive degradation of the normalized vasculature, which caused inefficient tumor penetration by cisplatin. Further research to determine the mechanism of Endostar and cisplatin synergy is required to clarify this point.

In conclusion, the results of the present study demonstrate that the simultaneous treatment of solid tumors with cisplatin and Endostar can effectively inhibit the growth of LLC xenografts, improve cell cycle distribution, increase cisplatin concentration in the tumor tissue and improve the vascular structure of the tumor. Therefore, the simultaneous, rather than the sequential administration of cisplatin and Endostar may be more effective for the treatment of tumors.

## Figures and Tables

**Table I:** Mean concentration of cisplatin in the tumor tissues and blood at various time-points following treatment with different drug schedules, as determined by high-performance liquid chromatography.

	Concentration of DDP, μg/ml							
	
	Day 1		Day 3		Day 5		Day 8	
				
Groups	Tissue	Blood	Tissue	Blood	Tissue	Blood	Tissue	Blood
DDP	1.073±0.116	2.077±0.274	1.027±0.024	2.019±0.319	0.845±0.211	0.964±0.189	1.680±0.323	2.174±0.214
Endo+DDP	1.173±0.119	2.274±0.255	2.666±0.255	2.219±0.355	1.357±0.153	0.736±0.175	3.794±0.210	2.932±0.341
Endo first	1.078±0.210	2.433±0.276	1.047±0.260	2.235±0.302	1.028±0.219	1.173±0.192	1.307±0.293	2.710±0.410

Mean ± standard deviation (n=5). DDP, cisplatin.

**Table II:** Mean tumor MVD at various time-points following treatment with different drug schedules.

	MVD (×400)			
	
Groups	Day 1	Day 3	Day 5	Day 8
DDP	5.50±0.71	6.83±0.29	8.10±1.15	10.00±1.02
Endo+DDP	5.33±0.58	6.33±0.58	5.50±0.71	4.23±0.40
Endo first	4.67±0.58	6.43±0.51	6.00±1.00	5.57±0.58

Mean ± standard deviation (n=5). MVD, microvessel density. DDP, cisplatin.
